# Effects of diclofenac on ventricular muscle repolarization: proarrhythmic implications

**DOI:** 10.1186/1471-2210-11-S2-A59

**Published:** 2011-09-05

**Authors:** Norbert Jost, Attila Kristóf, Zsófia Kohajda, Tamás Szél, Zoltán Husti, István Koncz, Victor Juhász, István Baczkó, Julius Gy Papp, András Varró, László Virág

**Affiliations:** 1 Department of Pharmacology and Pharmacotherapy, University of Szeged; Division of Cardiovascular Pharmacology, Hungarian Academy of Sciences, 6720 Szeged, Hungary

## Background

The aim of the present work was to characterize the electrophysiological effects of the non-steroidal anti-inflammatory drug diclofenac and to study the possible proarrhythmic potency of the drug in ventricular muscle.

## Methods

Ion currents were recorded using the voltage clamp technique in canine ventricular cells, and action potentials (AP) were recorded from canine ventricular preparations using microelectrodes. The proarrhythmic potency of diclofenac was investigated in an anaesthetized rabbit proarrhythmia model.

## Results

Diclofenac (30 µM) decreased the amplitude of rapid (I_Kr_) and slow (I_Ks_) delayed rectifier and L-type calcium currents (I_Ca_) without influencing transient outward (I_to_) and inward rectifier (I_K1_) potassium currents. The action potential was slightly lengthened in ventricular muscle but shortened in Purkinje fibres by diclofenac (20 µM). The maximum upstroke velocity (V_max_) was decreased in both preparations. Larger repolarization lengthening was observed when repolarization reserve was impaired by previous BaCl_2_ application. Diclofenac (3 mg/kg) did not prolong the QT_c_ interval, while the potassium channel blocker dofetilide (25 µg/kg) significantly lengthened QT_c_ in anaesthetized rabbits. The combination of diclofenac and dofetilide significantly prolonged QT_c_. Diclofenac alone did not induce torsades de pointes ventricular tachycardia (TdP) while TdP incidence following dofetilide was 20%. However, the combination of diclofenac and dofetilide led to a significant increase in the incidence of TdP.

## Conclusions

The results indicate that diclofenac, at therapeutic concentration and even at high dose, does not increase the risk of arrhythmia in normal heart. However, high dose drug treatment may enhance the proarrhythmic risk in the heart when the repolarization reserve is reduced.

